# Cannabis Use in People With Obsessive-Compulsive Symptomatology: Results From a Mexican Epidemiological Sample

**DOI:** 10.3389/fpsyt.2021.664228

**Published:** 2021-05-10

**Authors:** Humberto Nicolini, José Jaime Martínez-Magaña, Alma Delia Genis-Mendoza, Jorge Ameth Villatoro Velázquez, Beatriz Camarena, Clara Fleiz Bautista, Marycarmen Bustos-Gamiño, Alejandro Aguilar García, Nuria Lanzagorta, María Elena Medina-Mora

**Affiliations:** ^1^Genomics Laboratory of Psychiatric and Neurodegenerative Diseases, National Institute of Genomic Medicine, Mexico City, Mexico; ^2^Juan N. Navarro Children's Psychiatric Hospital, Psychiatric Care Services, Mexico City, Mexico; ^3^Data Analysis and Survey Unit, Ramón de la Fuente Muñiz National Institute of Psychiatry, Mexico City, Mexico; ^4^Global Studies Seminar, Faculty of Medicine, National Autonomous University of Mexico, Mexico City, Mexico; ^5^Department of Pharmacogenetics, Ramón de la Fuente Muñiz National Institute of Psychiatry, Mexico City, Mexico; ^6^Carraci Family Medical Research Group, Mexico City, Mexico

**Keywords:** obsessive-compulsive symptoms, cannabis use, Mexican population, polygenic risk score, cannabis dependence

## Abstract

Recent studies suggest that the endocannabinoid system could play an important role in the physiopathology of obsessive-compulsive disorder (OCD). There are reports of effective treatment with derivatives of tetrahydrocannabinol (THC). The study of the genetic factor associated with psychiatric disorders has made possible an exploration of its contribution to the pharmacological response. However, very little is known about the genetic factor or the prevalence of cannabis use in the Mexican population with OCD. The objective of this study is to compare the prevalence of use and dependence on cannabis in individuals with obsessive-compulsive symptomatology (OCS) with that of individuals with other psychiatric symptoms (psychosis, depression, and anxiety), and to explore the association between genetic risk and use. The study includes a total of 13,130 individuals evaluated in the second stage of the 2016 National Survey of Drug, Alcohol, and Tobacco Use (Encodat 2016), with genetic analysis (polygenic risk scoring) of a subsample of 3,521 individuals. Obsessive symptomatology had a prevalence of 7.2% and compulsive symptomatology a prevalence of 8.6%. The proportion of individuals with OCS who had ever used cannabis was 23.4%, and of those with cannabis dependency was 2.7%, the latter figure higher than that in individuals with other psychiatric symptoms (hypomania, 2.6%; anxiety, 2.8%; depression, 2.3%), except psychosis (5.9%). Individuals with OCS who reported using cannabis had an increased genetic risk for cannabis dependence but not for OCD. We thus cannot know how the increased genetic risk of cannabis dependence in people with OCD is influenced by their pharmacological response to derivatives of THC. The results, however, suggest paths for future studies.

## Introduction

Obsessive-compulsive disorder (OCD) is a chronic disorder that affects 1–3% of the population worldwide (1–3). Its diagnosis is based on the presence of obsessions, compulsions, or both (4). The obsessions usually take the form of recurrent thoughts, impulses, or images that can cause anxiety, while the compulsions are repetitive behaviors or mental acts that respond to the obsession (5). People diagnosed with OCD have a high rate of comorbidity with other disorders: an estimated 75.0% present an additional during their lifetime (6). OCD is a symptomatological spectrum, which has made it difficult to clarify its etiology, but there are some known risk factors, including a genetic factor and alterations in neurotransmitters and brain function (7). Genes have recently been discovered that may play an important role in the alteration of glutamatergic signaling (8), and OCD is one of the mental disorders most accompanied by alterations in brain function, mainly in the cortical-striatal-thalamic circuit (9–11). Alterations have also been reported in the levels of neurotransmitters such as serotonin, dopamine, and glutamate (7).

Another neurotransmission system associated with OCD, which has gained importance as a target for the development of possible pharmacological treatments, has been the endocannabinoid system (12–15). Some studies have shown that treatment with derivatives of tetrahydrocannabinol (THC), a partial agonist of the cannabinoid B1 receptor, could diminish anxiety-related symptoms in individuals with post-traumatic stress (13, 14, 16). Currently, the main pharmacological treatment for OCD is serotonin reuptake inhibitors, and the search for new treatments and growing approval of THC derivatives has produced favorable results in case studies (17). However, we cannot disregard the relationship between cannabis use and the development of dependence (18–20), which is inheritable, complex, and associated with mental health disorders (21, 22). A recent study explored genetic factors associated with lifetime use of cannabis and found ~35 genes in a sample of more than 180,000 individuals (23). Cannabis use with dependence has increased in Mexico from 2.4% in 2008 to 5.2% in 2016 (24), and Mexico is known for cannabis production (25).

Neither the prevalence of OCD, its relationship with the use of and dependence on cannabis, nor the genetic risk factors have been estimated on a populational level in Mexico. The objective of this study is thus to compare the prevalence of use and dependence on cannabis in individuals with obsessive-compulsive symptomatology (OCS) with that in individuals with other psychiatric symptoms (psychosis, depression, and anxiety) in a populational sample, and to explore the genetic risks associated with its use.

## Materials and Methods

### Study Population

The study included a total of 13,130 Mexican respondents from the second stage of the National Survey on Drug, Alcohol, and Tobacco Use (Encuesta Nacional de Consumo de Drogas, Alcohol y Tabaco 2016; Encodat 2016). The Encodat 2016 is a household survey aimed at assessing the patterns of use of different psychoactive drugs and certain mental health problems in the Mexican population. The survey was cross-sectional, with a multi-stage, probabilistic, and stratified design, and a confidence level of 90%. The sampling universe for the primary sampling units (PSUs) was the sum of the Basic Geographical Statistical Areas (BGSAs), stratified according to state and urban-rural character. Participants were 12–65 years of age, from urban and rural communities, and living at home. Wherever possible, following the household questionnaire, one adult aged 18–65 and one teenager aged 12–17 were presented with the individual questionnaire, according to a simple random sampling in each age group. The Encodat 2016 was nationally representative, with a total response rate (household + individual) of 73.6% and a final sample of 56,877 complete interviews: 27,463 men and 29,414 women; 9,563 teenagers and 47,314 adults.

The sample was obtained in two representative blocks: two independent national samples. However, it was in the second national sample that the symptomatology screening section was included with the psychiatric standard questionnaire on alcohol, tobacco, and drugs. The latter was performed for those who agreed to provide a DNA sample, with the screening questionnaire of the Diagnostic Interview for Psychosis and Affective Disorders (DI-PAD screener, version 1.5) (26–28), which is based on the Diagnostic Interview for Genetic Studies and is linked to the Operational Criteria for Psychotic Illness (OPCRIT, version 4.0). The DI-PAD screener was applied by an interviewer with specialized training in its use. Of the total of 28,770 participants in this second sample, 13,130 agreed to provide a DNA sample (Table 1) and answer the screener questions. This sample was weighted to obtain data that were representative on the national level.

**Table 1:** Sociodemographic and clinical characteristics of the total sample.

	**MxGDAR *(n* = 13 130)**
Age, in years (mean, sd)	33.5 (15.3)
**Gender**	
Male	6295 (47.9)
Female	6835 (52.1)
**Marital status**	
Married	4565 (34.8)
Cohabiting	2352 (17.9)
Separated	546 (4.2)
Divorced	151 (1.1)
Widowed	313 (2.4)
Single	5203 (39.6)
**Religion**	
Catholic	10329 (78.7)
Protestant	173 (1.3)
Jewish	3 (0.0)
Christian	898 (6.8)
Other	541 (4.1)
None	1186 (9.0)
**Educational level**	
Incomplete elementary level	1109 (8.4)
Completed elementary level	1914 (14.6)
Incomplete middle school	1365 (10.4)
Completed middle school	3518 (26.8)
Incomplete high school	1287 (9.8)
Completed high school	1982 (15.1)
University or more	1627 (12.4)
**Psychiatric symptoms lifetime**	
Obsession and compulsion	288 (2.4)
Hypomania	1033 (7.9)
Psiychosis	251 (1.9)
Anxiety	510 (3.9)
Depression	839 (6.4)
Cannabis use lifetime	1368 (10.4)
Cannabis dependence lifetime	82 (0.6)

### DI-PAD Screener Definition of Psychiatric Symptomatology

Participants were evaluated for lifetime psychiatric symptomatology using the following questions regarding obsessive symptomatology (OS) and compulsive symptomatology (CS):

OS symptomatology: “Have you ever had repetitive thoughts or images, much more exaggerated than normal worries, that you couldn't get out of your head, that were intrusive and uncomfortable, and that lasted an hour or more a day?”

CS symptomatology: “Have you ever had to repeat certain behaviors over and over for an hour or more a day? (Examples: washing your hands or checking the locks over and over again, or repeating words or counting things in your head.)”

Obsessive-Compulsive symptomatology (SOC): affirmative response to both of the above.

Definitions for other psychiatric symptomatologies (psychosis, hypomania, anxiety, and depression) were as following:

1. Hypomania.

To define a case must meet the following criteria:

- Have you ever been diagnosed with bipolar disorder (or manic depressive disorder)?

If you do not meet the above criteria, must meet the following two criteria:

- Have you ever had a period of time that lasted 3 days or more in which you felt unusually cheerful, irritable, energetic, or hyperactive, so much so that you felt or acted in a way that was clearly different from your normal character?
- Have you ever had a period of time that lasted 3 days or more in which you didn't need much sleep (or no sleep at all) without feeling tired, or even had more energy than normal?

2. Psychosis.

To define a case must meet the following criteria:

- Have you ever been diagnosed with schizophrenia?
- If you do not meet the above criteria, must meet the following two criteria:
- Have you ever had a period of time when you heard voices when no one was actually present, had visions, or saw things that other people couldn't see?
- Have you ever had beliefs or ideas that other people did not share with you or that you later discovered were not true?

3. Anxiety.

To define a case must meet the following three criteria:

- Have you ever had an experience where you suddenly felt very anxious or fearful?
- When you had this experience, did you feel rapid heartbeat, chest pain, feeling short of breath or strangulation, nausea, sweating, weakness, thinking you would go crazy or die?
- Did these problems get worse or stronger in the first 10 min?

4. Depression.

To define a case must meet the following criteria:

- Have you ever felt depressed, sad, down, or discouraged most of the day, almost every day, for 2 weeks or more?

In addition, must meet at least one of the following criteria:

- Have you had a period of 2 weeks or more in which you lost most or all interest in your normal activities?
- During this period, did you also have feelings of worthlessness or guilt, or did you spend a lot of time with thoughts of death, suicide, or self-harm?
- During this period, did you notice a significant change in your appetite, unexpected weight gain or loss, experienced changes in your normal sleep pattern, or had difficulty concentrating?

The use of or dependence on cannabis was evaluated in this study under the criteria of the Composite International Diagnostic Interview (CIDI). Dependence was considered to be three or more of the following symptoms: tolerance, abstinence, a longer time or a greater amount of use, persistent or uncontrollable cravings, excessive time spent in getting drugs or recuperating from their effects, reduction in social, work, or recreational activities, or continued use in spite of awareness of harmful effects.

All participants provided written informed consent or assent. The protocols were carried out based on international norms and the Helsinki Declaration; they were reviewed and approved by the research and ethics committees of the Instituto Nacional de Psiquiatría (Approval No. CEI/C/083/2015) and the Instituto Nacional de Medicina Genómica (Approval No. 01/2017/I).

### Microarray Analysis

DNA was collected from cheek swabs, using a modified salting-out method with the Puregen commercial kit (Qiagen, USA). The quality and integrity of the DNA was evaluated with a NanoDrop spectrophotometer (Thermofisher, USA) and a 2% agarose gel. The procedure produced 7,170 samples of sufficient quality for microarray analysis. The genotyping was carried out with an Infinium Psycharray commercial microarray (Illumina, USA). The fluorescence intensities were read with iScan (Illumina, USA). The genotyping procedure was carried out in the high-technology microarray unit of the National Institute of Genomic Medicine. Genotyping was performed on a subsample of 3,600 individuals. A random sampling of the 7,170 samples was performed to select 60% with psychiatric symptomatology and 40% as controls.

### Calling and Quality Control of Single Nucleotide Polymorphisms (SNPs)

The fluorescence intensities were transformed to genotypes using the software GenomeStudio (Illumina, USA), and quality control was performed with the Plink program (29). Single nucleotide variants (SNVs) with a call rate >95% were removed, as were those with a minor allele frequency (MAF) >1%, a *p*-value > 1e-6 for a chi-square Hardy-Weinberg equilibrium test, and A/T or G/C variants (to avoid the flip strand effect). Individuals with a genotyping of <95% were removed. To correct cryptic relationships, all pairs of individuals with an identity-by-state value >1.6 were marked, and the individual with the lowest rate of genotyping was removed (30).

### Statistical Analyses

To evaluate the genetic risk for obsessive-compulsive disorder and lifetime use of cannabis, polygenic risk scores (PRS) were calculated, using summary statistics for cannabis dependence (23) and the scores reported by the Psychiatric Genomic Consortium for obsessive-compulsive disorder (8). Polymorphisms were selected with *p*-values < 0.05, as reported in the summary statistics, which had good genotyping quality control. The PRS were correlated with the principal components of ancestry. Genetic ancestry was estimated using principal component analysis with the PC-AiR package (31) and the reference base of the Human Genome Diversity Project (32). The standardized residuals for comparison between groups were obtained based on the correlations of the PRS with ten principal components of genetic ancestry. The comparisons of the PRS were performed using ANOVA or Student's *t*-test.

## Results

### Estimation of the Prevalence of Obsessive-Compulsive Symptomatology

The prevalence of obsessive symptomatology (OS) was 7.1% (*n* = 866), of compulsive symptomatology (CS) was 8.2% (*n* = 1,004), and of obsessive-compulsive symptomatology (OCS) was 2.4% (*n* = 288). Differences were found in the presence of OCS between men (42.3%) and women (57.7%), but these were not statistically significant compared with gender differences in those without OCS (*?*^2^ = 1.4, *p* = 0.8620). The average age of those with OCS was less (*m* = 30.1, *SD* = 15.4) than those without such symptomatology (*m* = 33.4, *SD* = 15.7) (*T* = −3.9, *p* < 0.0001).

### Estimation of Cannabis Use in Individuals With Obsessive-Compulsive Symptomatology

**Figure 1:**
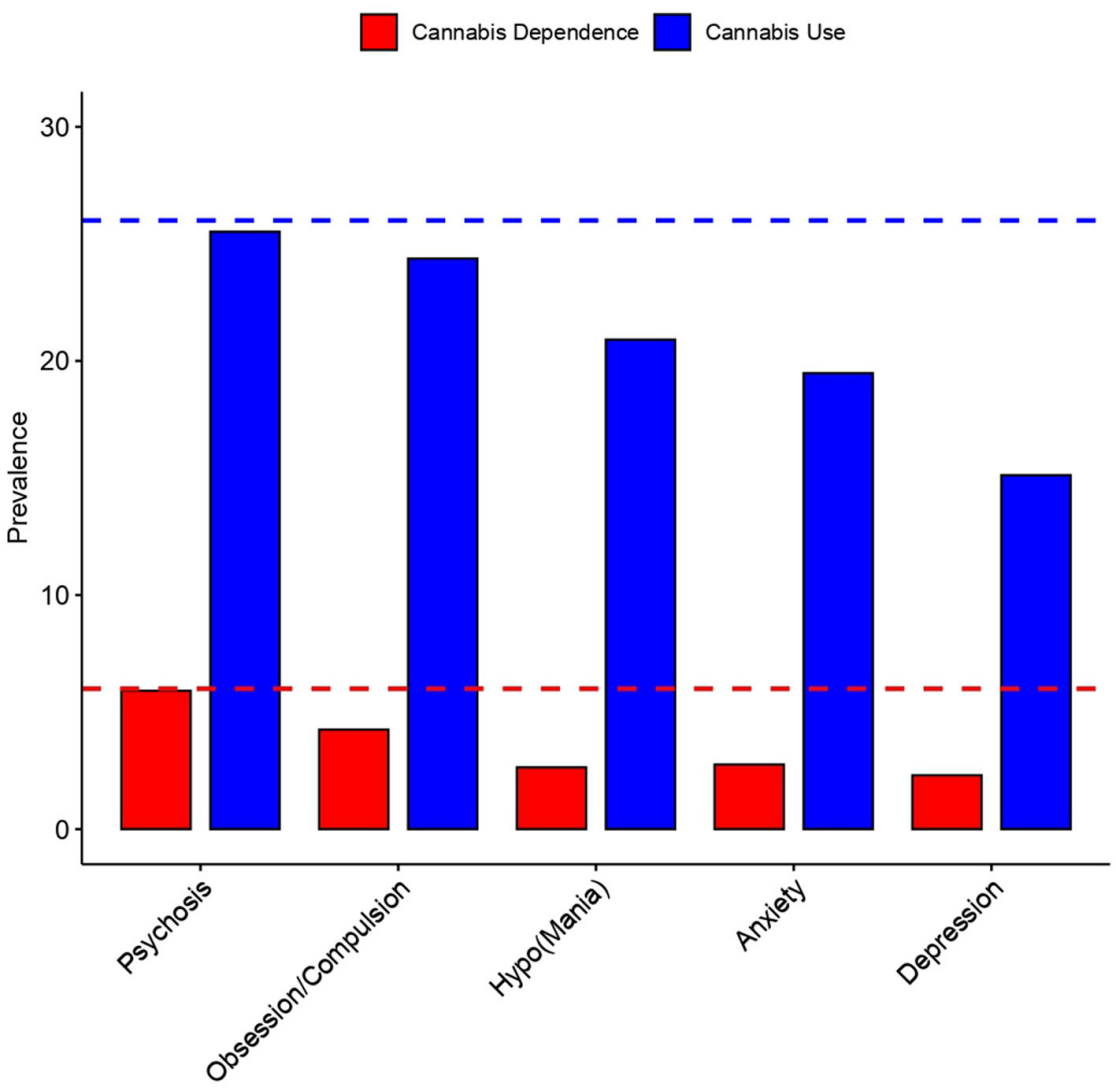
Prevalence of Cannabis use and dependence in the population. Cannabis dependence is shown in red, and cannabis use in blue. The prevalence was divided in the psychiatric symptomatology.

The prevalence of having ever used cannabis was 24.4% (*n* = 70) in individuals with OCS (*n* = 288), greater than that reported for the population as a whole (9.7%) and greater than that for those with other psychiatric symptoms (hypomania, 20.9%; anxiety, 19.5%; and depression, 15.1%) except psychosis (25.5%) (Figure 1). The prevalence of having ever used cannabis was similar in those with only OS (15.8%, *n* = 149) as in those with only CS (15.2%, *n* = 172).

### Estimation of Cannabis Dependence in Individuals With Obsessive-Compulsive Symptomatology

The prevalence of having ever been dependent on cannabis in individuals with OCS was greater (4.3%, *n* = 12) than that found in the population as a whole (0.6%). Greater dependence was also observed in this group than in individuals with other psychiatric symptomatologies (hypomania, 2.6%; anxiety, 2.8%; depression, 2.3%) except psychosis (5.9%). However, dependence was greater in individuals with only CS (2.2%, *n* = 22) than in those with only OS (1.8%, *n* = 15).

### Comparison of the Genetic Risk in Individuals With OCS for OCD in the Use of Cannabis

**Figure 2:**
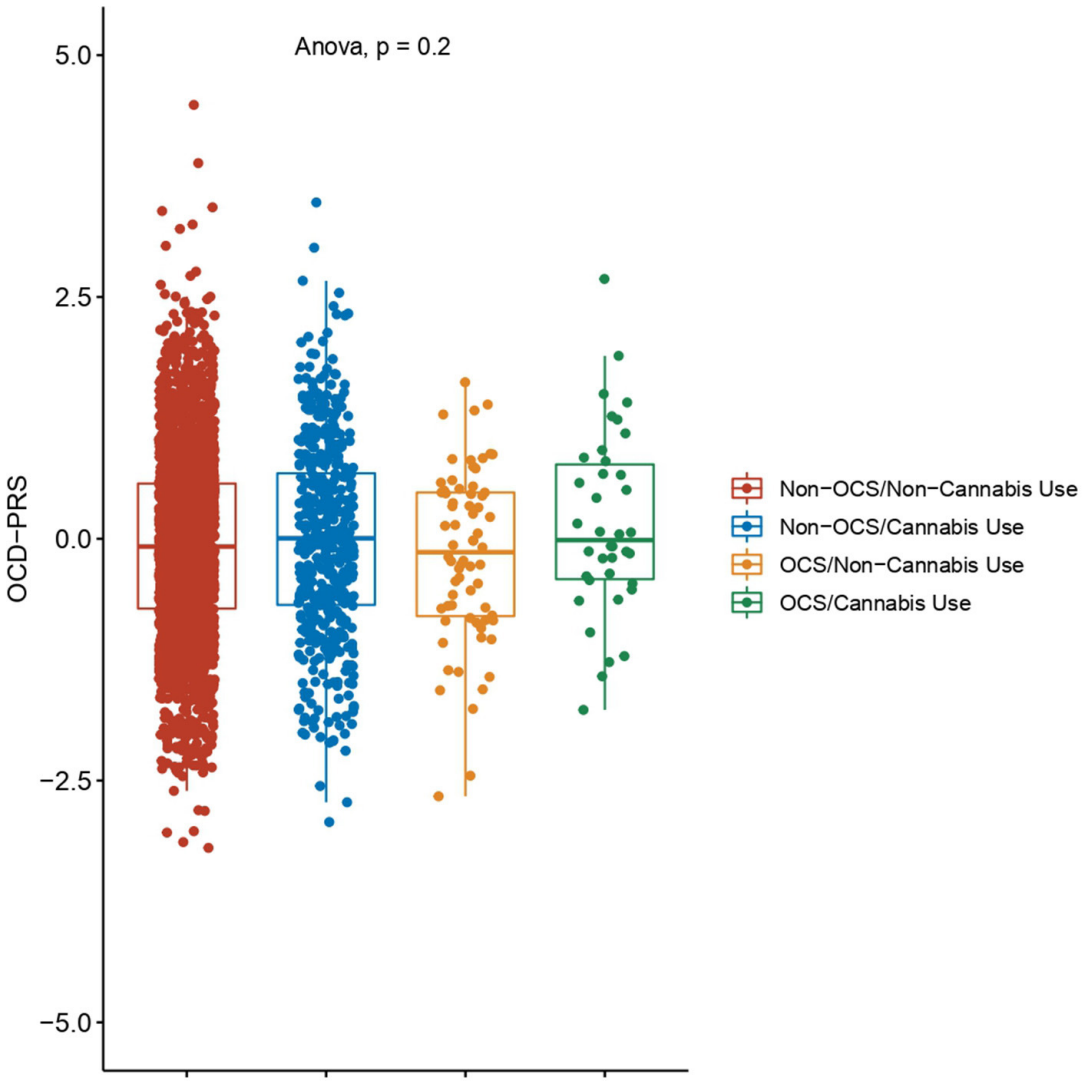
Polygenic risk score for obsessive-compulsive disorder (OCD-PRS). The OCD-PRS was calculated with data reported by the Psychiatric Genomics Consortium and standardized by ancestry. Differences between the four groups were evaluated by ANOVA. Non-OCS: individuals without OCS symptoms; Non-Cannabis Use: individuals who had never used cannabis; OCS: individuals with OCS symptoms; and Cannabis Use; individuals who had ever used cannabis.

The subsample used for genotyping had the following distribution: 81.8% (*n* = 2,658 individuals) with no cannabis use or OCS, 14.9% (*n* = 485 individuals) having ever used cannabis but with no OCS, 2.1% (*n* = 68 individuals) with OCS but no cannabis use, and 1.2% (*n* = 38 individuals) with OCS and having ever used cannabis. The PRS for OCD was constructed using 11,959 SNPs, which passed the quality control test. The OCD-PRS comparison found no statistically significant differences between the different groups (*F* = 1.5, *p* = 0.2020) (Figure 2).

**Figure 3:**
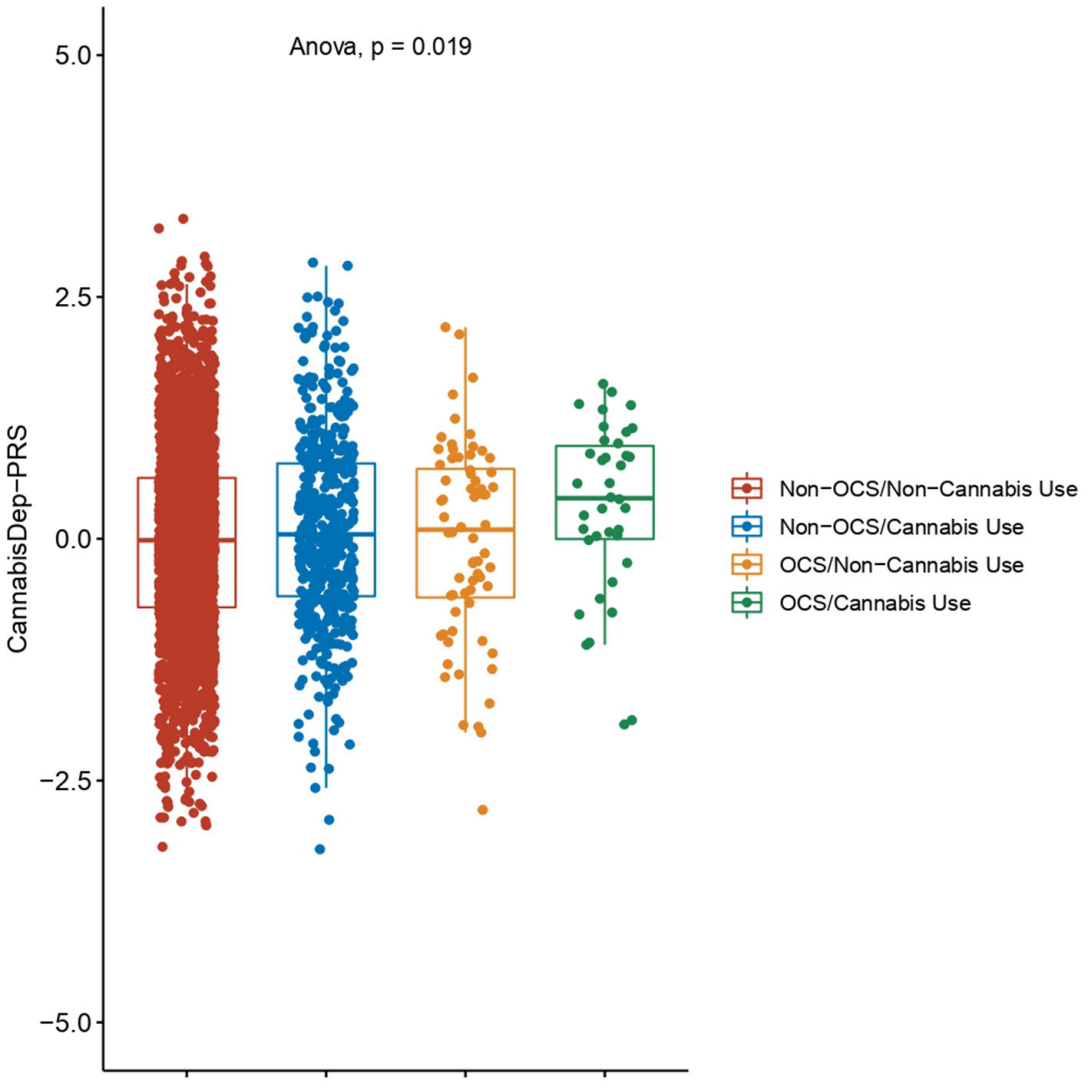
Polygenic risk score for cannabis dependence (CannabisDep-PRS). The CannabisDep-PRS was calculated with data reported from the Psychiatric Genomics Consortium and standardized by ancestry. Differences between the four groups were evaluated by ANOVA. Non-OCS: individuals without OCS symptoms; Non-Cannabis Use: individuals who had never used cannabis; OCS: individuals with OCS symptoms; and Cannabis Use: individuals who had ever used cannabis.

The PRS for dependence on cannabis was constructed using 13,485 SNPs, which passed the quality control test. The CannabisDep-PRS comparison found statistically significant differences between the different groups (*F* = 3.3, *p* = 0.0192) (Figure 3). The *post-hoc* comparisons found that the CannabisDep-PRS between individuals with OCS with cannabis use were not significantly different from those of individuals with OCS but with no cannabis use (*p* = 0.2010). Individuals with OCS and cannabis use had the highest CannabisDep-PRS value (*m* = 0.3151), and the difference from the value for those without OCS or cannabis use was statistically significant (*p* = 0.0390).

## Discussion

Cannabis dependence and use has increased in recent years, as have proposals for the use of THC derivatives as pharmacological agents in areas such as psychiatry and oncology (33–36). In psychiatry, these derivatives could improve the symptomatology of some disorders, mainly those related to stress, such as Tourette's syndrome, anxiety, and post-traumatic stress disorder (16, 22, 37–40). Even a non-psychoactive derivative (cannabidiol) has been used to treat substance use disorders (41, 42). There are case studies that suggest a possible improvement in OCD after treatment with THC derivatives (17). A pilot clinical trial found that use of THC derivatives with exposure therapy has a synergic effect on the treatment of obsession and compulsion on individuals diagnosed with OCD (43), and its use as a therapeutic agent should still be explored. But we cannot disregard the effect of the continuous use of cannabis on the development of cannabis use disorder where its use is already problematic. We found that the prevalence of cannabis dependence in individuals with obsessive-compulsive symptomatology (OCS) was second (at 4.3%) only to its prevalence in those with psychosis (5.9%) (44). This prevalence in those with OCS is interesting, given that psychosis, but not OCD, has been strongly associated with use of or dependence on cannabis (45). Such an association has been found for other psychiatric disorders, including depression, anxiety, and suicide attempts, and for psychosis manifested at earlier ages (45–48). We do not, however, know of its effect on OCS or on the development of psychosis.

One of the important aspects to consider in the relationship between cannabis and OCD is the development of cannabis dependence either through recreational use or in pharmacological applications. This development has been thought of as a complex phenotype that must include different risk factors, such as the genetic one, in its development (49–51). The genome scans have found hundreds or thousands of associated genetic variations that have been used to calculate polygenic risk scores (PRS) (52–55). We have found that the PRS for cannabis dependence was greater in those with OCD who used cannabis than in those who did not use it. Individuals with a high genetic risk who use cannabis might thus increase their risk of dependence either through recreational use or with pharmacological derivatives of THC. PRS have been used not only as predictors of risk, but also as markers of pharmacological response to psychoactive agents and in pharmacogenomic studies (56–59). It might be hypothesized that the use of the PRS for cannabis dependence could be useful in predicting which individuals are at high risk, and to determine whether pharmacological treatment based on THC derivatives would be useful, or would exacerbate obsessive-compulsive symptomatology.

## Study Limitations

Although we found associations between obsessive-compulsive symptomatology and the use of or dependence on cannabis, a limitation of this study is its lack of direct psychiatric diagnostic evaluation of the individuals surveyed. A further limitation was our inability to conduct a longitudinal evaluation of OCD symptoms, before or after cannabis use, to evaluate the symptomatological changes brought about by that use. Finally, because of the sample size, our analysis of genetic risk was able to evaluate only the use of cannabis, and not dependence.

## Conclusions

The use of and dependence on cannabis was found to be greater in the Mexican population among individuals with obsessive-compulsive symptomatology than in those with anxiety or depression, but less than in those with psychosis. The genetic risk for cannabis dependence was also associated with cannabis use in individuals with obsessive-compulsive symptomatology. It may be possible in future pharmacogenomic studies to determine the response rates of individuals with different genetic risk scores.

## Data Availability Statement

The genomic datasets generated for this study can be found in the European Variation Archive (EVA, PRJEB37766).

## Ethics Statement

The studies involving human participants were reviewed and approved by the research and ethics committees of the Instituto Nacional de Psiquiatría Ramón de la Fuente Muñiz and the Instituto Nacional de Medicina Genómica. Written informed consent was provided by the participants or their parents or legal guardians.

## Author Contributions

HN, NL, JM-M, AG-M, and JV developed the analyses and wrote the first version of the manuscript. JM-M, JV, and MB-G performed bioinformatics and statistical analyses. JV, CF, MB-G, and MM-M contributed to data collection. BC, AA, JM-M, and AG-M contributed to the genetic experiments. HN, MM-M, and JV conceived, designed, and coordinated the genetic analysis. All authors contributed to the article and approved the submitted version.

## Conflict of Interest

The authors declare that the research was conducted in the absence of any commercial or financial relationships that could be construed as a potential conflict of interest.
